# Risk Factors for Disability Pension among Young Adults Diagnosed with Attention-deficit Hyperactivity Disorder (ADHD) in Adulthood

**DOI:** 10.1177/10870547211025605

**Published:** 2021-06-22

**Authors:** Lingjing Chen, Ellenor Mittendorfer-Rutz, Emma Björkenstam, Syed Rahman, Klas Gustafsson, Heidi Taipale, Antti Tanskanen, Lisa Ekselius, Magnus Helgesson

**Affiliations:** 1 Karolinska Institutet, Stockholm, Sweden; 2 Uppsala University, Sweden; 3 Niuvanniemi Hospital, Kuopio, Finland; 4 University of Eastern Finland, Kuopio, Finland

**Keywords:** ADHD, disability pension, young adults, register data

## Abstract

**Objective:**

To investigate risk factors of disability pension (DP) in young adults diagnosed with ADHD in Sweden.

**Method:**

In total, 9718 individuals diagnosed with incident ADHD in young adult age (19–29 years) 2006 to 2011, were identified through national registers. They were followed for 5 years and Cox regression models were applied to analyze the DP risk (overall and by sex), associated with socio-demographics, work-related factors, and comorbid disorders.

**Results:**

Twenty-one percent of all received DP. Being younger at diagnosis (hazard ratio [HR] = 1.54; 95%confidence interval [CI] 1.39–1.71); low educational level (HR = 1.97; 95%CI 1.60–2.43 for <10 years); work-related factors at baseline (no income from work [HR = 2.64; 95%CI 2.35–2.98] and sickness absence >90 days [HR = 2.48; 95%CI2.17–2.83]); and schizophrenia/psychoses (HR = 2.16; 95%CI 1.66–2.80), autism (HR = 1.87; 95%CI 1.42–2.46), anxiety (HR = 1.34; 95%CI 1.22–1.49) were significantly associated with an increased risk of DP. Similar risk patterns were found in men and women.

**Conclusion:**

Work-related factors and comorbid mental disorders need to be highlighted in early vocational rehabilitation for individuals with ADHD.

## Background

As a neurodevelopmental disorder, ADHD is usually diagnosed in childhood and may further persist into adulthood (Rydell et al., 2018; Thapar & Cooper, 2016; Thomas et al., 2015). The prevalence of ADHD in an adult population is estimated to be around 3.6% in high-income countries, based on the World Mental Health Surveys (Fayyad et al., 2017). During the recent years, the number of young adults diagnosed with ADHD is increasing, both internationally and in Sweden (Chung et al., 2019; Giacobini et al., 2018; Polyzoi et al., 2018), making ADHD a considerable public health concern.

ADHD has far-reaching negative impacts on the affected individuals’ well-being and social integration, posing a great societal burden (Coghill et al., 2017; Cortese & Coghill, 2018; de Graaf et al., 2008; Fredriksen et al., 2014; Halmoy et al., 2009; Virtanen et al., 2020). For example, ADHD may lead to functional impairment (Coghill et al., 2017), higher risks of injuries and accidents (Küpper et al., 2012), which in turn may decrease one’s work ability (de Graaf et al., 2008; Fredriksen et al., 2014; Halmoy et al., 2009; Küpper et al., 2012). According to a recent Swedish study of 3 years’ follow-up, compared with the general population, young adults with an ADHD diagnosis had nearly a 12-fold risk of work disability (sum of sickness absence and disability pension days), compared with a matched national cohort (Virtanen et al., 2020). Meanwhile, other mental disorders, such as mood and anxiety disorders, and other common mental disorders, are common comorbidities of ADHD in young adults (Chung et al., 2019; Gnanavel et al., 2019; Katzman et al., 2017; Sobanski, 2006; Thapar & Cooper, 2016). These mental disorders of ADHD are found to be associated with a heightened risk of work disability (Helgesson et al., 2017, 2018).

Previous studies suggested that sociodemographic factors (such as educational level, type of living area) (Virtanen et al., 2020), and comorbid mental disorders (depression and anxiety disorders) (Fredriksen et al., 2014; Halmoy et al., 2009) may be associated with a higher risk of work disability in persons with ADHD later in life. Gender differences in the association between ADHD and work disability also warrants further attention. Among individuals diagnosed with ADHD, women often present fewer symptoms of hyperactivity, inattention, impulsivity, and externalizing problem (Gershon & Gershon, 2002). Instead, they tend to have internalizing problems, leading to a high risk of comorbid conditions such as depression and anxiety (Gershon & Gershon, 2002). Meanwhile, gender differences in sickness absence and disability pension have since long been established in occupational health (Laaksonen et al., 2010), and are therefore important aspects to be considered in the research of ADHD and work disability.

Work participation among young adults with ADHD diagnosis should be closely monitored and followed in combination with unfavorable baseline work-related factors, in order to prevent these individuals from being stuck in a state of labor market marginalization permanently. So far, there is very little research on the relationship between ADHD and young adults’ future work disability. The extent of permanent isolation from the labor market for these individuals remains unknown. The few existing studies are often only based on small study populations and short follow-up (Fredriksen et al., 2014; Halmoy et al., 2009; Virtanen et al., 2020). Therefore, high quality population-based register studies that investigate long-term work capacity longitudinally among these young adults are highly warranted.

Presently, we employed a large cohort study based on longitudinal Swedish register data, in order to inform clinicians of factors that are associated with a greater risk of disability pension in patients with ADHD and those patients that may benefit from early vocational interventions. Specifically, we aimed to investigate the associations between disability pension and factors including sociodemographic and work-related ones, and comorbid disorders, in individuals with ADHD. Further, we studied these associations separately in men and women.

## Methods

### Study Population

Using data from Swedish national registers, we identified all 12,776 young adults between 19 and 29 years of age with an incident diagnosis of ADHD from specialized health care between January 2006 and December 2011. The day of diagnosis was defined as cohort entry date (CED). The diagnosis of ADHD was defined as having the diagnostic code (F90) from the International Classification of Diseases, 10th Revision (ICD-10). Those who had an ongoing disability pension during the year of their ADHD diagnosis year were excluded (*n* = 3,058). The final study population consisted of 9,718 people. The project was approved by the Regional Ethical Review Board of Stockholm, Sweden.

### National Registers

Microdata were obtained from five national registers and merged at an individual level using the personal identification number given to all residents in Sweden (Ludvigsson et al., 2009).

*From Statistics Sweden*: the longitudinal Integration Database for Health Insurance and Labor Market Studies (LISA) (Ludvigsson et al., 2019) (sociodemographic information: sex, year of birth, educational level, region of birth, family composition, type of living area, year of emigration; work-related information; income from work, unemployment).

*From the National Board of Health and Welfare*: the Prescribed Drug Register (Wettermark et al., 2007) (for purchases of prescribed medications for diabetes); the National Patient Register (Ludvigsson et al., 2011) primary and secondary diagnoses for ADHD and all comorbid disorders during the study period; the Cause of Death Register (for death date) (Brooke et al., 2017).

*From the Swedish Social Insurance Agency (SSIA)*: The Microdata for Analyses of Social Insurance (MiDAS) (Österlund, 2011), all baseline sickness absence >14 days (for the employed) and disability pension regarding: date, duration and grade.

### Outcome Measure

The outcome was the granting of disability pension within a 5-year period after the first ADHD diagnosis in adult age.

Time-restricted disability pension can be granted to individuals between 19 and 29 years of age, given that their work capacity is reduced, or compulsory education is not finished when they become 19 years old. Thereafter, if the same conditions remain, the temporary disability pension will become permanent when they turn 30 years of age (Swedish Social Insurance Agency, 2012). Disability pension may be granted at four levels in relation to full working time (25%, 50%, 75%, and 100%). The grade of disability pension is based on individuals’ health conditions and work capacity assessed by certified physicians.

### Covariates

Three sets of individual characteristics were investigated: (1) socio-demographic factors including sex, age, educational level, region of birth, family composition, and type of living area; (2) work-related factors including having income from work, unemployment status, and sickness absence status at baseline (socio-demographic and work-related factors were measured during the year before the year of ADHD diagnosis); and (3) comorbid disorders based on the primary and secondary diagnoses of inpatient and specialized outpatient health care during 1 year before their ADHD diagnosis dates. The Prescribed Drug Register was used to ascertain the diagnosis of diabetes.

The comorbid diagnoses were categorized as any mental (depression and bipolar disorders, anxiety and stress-related disorders, autism-spectrum disorders, substance abuse, behavioral and emotional disorders, schizophrenia/psychotic disorders, mental retardation, and other mental disorders) and any somatic (musculoskeletal disorders and other somatic disorders) diagnoses respectively. The detailed categorization of different covariates and the classification of comorbid diagnoses is presented in Table 1.

**Table 1.** Characteristics of Patients with ADHD, Diagnosed in Specialized Health Care 2006 to 2011 (*n* = 9,718).

	*N* (%)
Sociodemographic factors	
Sex	
Female	4,166 (42.9)
Male	5,552 (57.1)
Age at diagnosis	
19–24 years (young)	6,037 (62.1)
25–29 years (old)	3,681 (37.9)
Educational level (years)^a^	
Low (<10)	4,665 (48.0)
Medium (10–12)	4,211 (43.3)
High (>12)	842 (8.7)
Region of birth	
Sweden	8,948 (92.1)
Nordic countries	73 (0.8)
EU27	100 (1.0)
Rest of the world	597 (6.1)
Family composition	
Married/cohabiting without children	116 (1.2)
Married/cohabiting with children	744 (7.7)
Single without children	8,318 (85.6)
Single with children	540 (5.6)
Type of living area^b^	
Big cities	3,329 (34.3)
Medium-sized cities	3,567 (36.7)
Small towns	2,822 (29.0)
Work-related factors	
Income from work	
Yes	6,264 (64.5)
No	3,454 (35.5)
Unemployment at baseline	
0 days	5,124 (52.7)
1–180 days (short)	3,358 (34.6)
>180 days (long)	1,236 (12.7)
Sickness absence at baseline	
0 days	8,142 (83.8)
1–90 days (short)	660 (6.8)
>90 days (long)	916 (9.4)
Comorbid diagnoses	
Depression and/or bipolar disorders (ICD-10^c^ F30–F34)	1,688 (17.4)
Anxiety and/or stress-related disorders (ICD-10: F40–F48)	2,154 (22.2)
Autism-spectrum disorders (ICD-10: F84)	144 (1.5)
Substance abuse (ICD-10: F10–F19)	1,518 (15.6)
Behavioral and/or emotional disorders (ICD-10: F91–F98)	224 (2.3)
Schizophrenia/psychoses (ICD-10: F20–F29)	129 (1.3)
Mental retardation^d^ (ICD-10: F70–F83, F85–F89)	59 (0.6)
Other mental disorders (ICD-10: Other F)	1,011 (10.4)
Musculoskeletal disorders (ICD-10: M00–M99)	490 (5.0)
Other somatic disorders (ICD-10: all remaining codes^e^)	4253 (43.8)

a <10 years is equivalent to elementary school; 10 to 12 years is equivalent to upper and secondary school; and >12 years is equivalent to college/university level.

b Big cities included: Stockholm, Göteborg and Malmö; medium-sized cities: cities with more than 90,000 inhabitants within 30 km distance from the center of the city; otherwise small cities/villages.

c International Classification of Diseases, Version 10 code.

d Disorders of psychological development.

e Except for ICD-10 codes O80 (single delivery) and Z00–Z99 (factors influencing health status and contact with health services).

### Statistical Analyses

The distribution of different individual characteristics in the study population was illustrated by descriptive analyses. A Cox proportional hazard regression (with a module of competing risks) was applied to study the associations between the risk of disability pension and different covariates. Hazard ratios (HRs) with 95% confidence intervals (CIs) were calculated. All included individuals were followed 5 years from the day after CED, until they were granted disability pension, emigrated from Sweden, died or the end of follow-up (December 31, 2016), whichever came first.

Besides applying a crude model to analyze the associations between individual characteristics and the risk of disability pension, we further performed three adjusted models: Model 1, with adjustment for sex, age, educational level, family composition, type of living area and region of birth; Model 2, with Model 1 and additional adjustment for unemployment, income from work and sickness absence at baseline; Model 3, the same as Model 2 with further adjustment for comorbid mental and/or somatic disorders. In order to study the sex-specific associations, we stratified the abovementioned analyses regarding sex. All statistical analyses were performed using SAS, version 9.4 (SAS Institute Inc.).

## Results

The majority of persons diagnosed with ADHD in the study were men, between the age of 19 and 24 and born in Sweden (Table 1). At baseline, nearly 65% had some income from work and over 80% did not have any sickness absence. The most prevalent comorbid mental disorders were anxiety and/or stress-related disorders, depression and/or bipolar disorders, and substance abuse.

Within 5 years, 19% of men and 25% of women (21% of all, *n* = 2,075) diagnosed with ADHD received disability pension (Table 2). Compared with men, women had 28% higher risk of being granted disability pension. People who received an ADHD diagnosis at the age of 19 to 24 had a significantly higher risk of being granted disability pension (HR = 1.54, 95%CI 1.39–1.71), compared with those diagnosed between 25 and 29. Compared with having high educational level, the risk of disability pension was 1.47 (95% CI 1.19–1.82) for those who had medium educational level, and 1.97 (95% CI 1.60–2.43) for those with low educational level. Compared with being single without children, being married and cohabiting with children was associated with a lower risk of disability pension in the future (HR = 0.74, 95%CI 0.61–0.91).

**Table 2.** Hazard ratios (HRs) With 95% Confidence Interval (CI) for Disability Pension in Association with Sociodemographic, Work-Related Factors and Comorbid Diagnoses Among Patients with Diagnosed ADHD (*n* = 9,718).

	Number of DP (% of all people in the corresponding stratum)	Crude model	Model 1^a^	Model 2^b^	Model 3^c^
	*n* (%)	HR (95% CI)	HR (95% CI)	HR (95% CI)	HR (95% CI)
Sociodemographic factors					
Sex					
Male	1,037 (18.68)	1	1	1	1
Female	1,038 (24.92)	**1.37 (1.26–1.50)**	**1.42 (1.30–1.56)**	**1.35 (1.23–1.48)**	**1.28 (1.17–1.41)**
Age at diagnosis					
19–24 years (young)	1,489 (24.66)	**1.62 (1.47–1.78)**	**1.41 (1.27–1.56)**	**1.55 (1.40–1.73)**	**1.54 (1.39–1.71)**
25–29 years (old)	586 (15.92)	1	1	1	1
Educational level (years)^d^					
Low (<10)	1,204 (25.81)	**2.35 (1.92–2.88)**	**2.19 (1.78–2.69)**	**1.89 (1.54–2.33)**	**1.97 (1.60–2.43)**
Medium (10–12)	769 (18.26)	**1.57 (1.28–1.93)**	**1.49 (1.21–1.84)**	**1.45 (1.18–1.79)**	**1.47 (1.19–1.82)**
High (>12)	102 (12.11)	1	1	1	1
Region of birth					
Sweden	1,912 (21.37)	1	1	1	1
Nordic countries	14 (19.18)	0.95 (0.56**–**1.61)	0.98 (0.58**–**1.66)	1.01 (0.60**–**1.71)	0.97 (0.57**–**1.64)
EU27	20 (20.00)	0.92 (0.59**–**1.43)	1.03 (0.67**–**1.61)	0.99 (0.64**–**1.54)	0.97 (0.62**–**1.51)
Rest of the world	129 (21.61)	1.03 (0.86**–**1.23)	1.08 (0.91**–**1.30)	1.06 (0.88**–**1.26)	1.06 (0.88**–**1.27)
Family composition					
Married/cohabiting without children	31 (26.72)	1.29 (0.90**–**1.84)	1.41 (0.99**–**2.02)	1.27 (0.89**–**1.82)	1.29 (0.90**–**1.85)
Married/cohabiting with children	112 (15.05)	**0.66 (0.54–0.80)**	**0.70 (0.58–0.85)**	**0.72 (0.59–0.88)**	**0.74 (0.61–0.91)**
Single without children	1,801 (21.65)	1	1	1	1
Single with children	131 (24.26)	1.13 (0.94**–**1.35)	0.99 (0.82**–**1.19)	0.96 (0.80**–**1.16)	1.01 (0.84**–**1.22)
Type of living area^e^					
Big cities	67 (19.13)	1	1	1	1
Medium-sized cities	779 (1.84)	**1.16 (1.04–1.28)**	**1.14 (1.02–1.26)**	1.05 (0.94**–**1.17)	1.03 (0.93**–**1.15)
Small towns	659 (23.35)	**1.25 (1.12–1.39)**	**1.20 (1.07–1.34)**	1.11 (0.99**–**1.24)	1.09 (0.98**–**1.22)
Work-related factors					
Income from work					
Yes	395 (11.44)	1	1	1	1
No	1,680 (26.82)	**2.61 (2.34–2.91)**	**2.33 (2.08–2.61)**	**2.66 (2.36–3.00)**	**2.64 (2.35–2.98)**
Unemployment at baseline					
0 days	1,056 (20.61)	1	1	1	1
1–180 days (short)	681 (20.28)	**1.38 (1.22–1.56)**	0.93 (0.84**–**1.02)	**0.84 (0.76–0.92)**	**0.84 (0.76–0.93)**
>180 days (long)	338 (27.35)	0.98 (0.89**–**1.08)	**1.31 (1.16–1.49)**	1.01 (0.89**–**1.14)	1.02 (0.90**–**1.16)
Sickness absence at baseline					
0 days	1,654 (20.31)	1	1	1	1
1–90 days (short)	97 (14.70)	0.70 (0.57**–**0.86)	0.83 (0.67**–**1.02)	**1.24 (1.01–1.54)**	1.12 (0.90**–**1.38)
>90 days (long)	324 (35.37)	**1.93 (1.72–2.18)**	**2.34 (2.07–2.65)**	**2.92 (2.57–3.32)**	**2.48 (2.17–2.83)**
Comorbid diagnoses					
Depression and/or bipolar disorders (ICD-10 F30–F34)	477 (28.26)	**1.50 (1.35–1.66)**	**1.53 (1.38–1.70)**	**1.38 (1.24–1.53)**	**1.22 (1.09–1.36)**
No depression nor bipolar disorders	1,598 (19.90)	1	1	1	1
Anxiety and/or stress-related disorders (ICD-10: F40–F48)	622 (28.88)	**1.60 (1.46–1.76)**	**1.60 (1.46–1.76)**	**1.46 (1.33–1.61)**	**1.34 (1.22–1.49)**
No anxiety nor stress-related disorders	1,453 (19.21)	1	1	1	1
Autism-spectrum disorders (ICD-10: F84)	54 (37.50)	**2.04 (1.56–2.68)**	**2.15 (1.64–2.83)**	**1.94 (1.48–2.54)**	**1.87 (1.42–2.46)**
No autism-spectrum disorders	2,021 (21.11)	1	1	1	1
Substance abuse (ICD-10: F10–F19)	370 (24.37)	**1.23 (1.10–1.37)**	**1.19 (1.06–1.33)**	1.11 (0.99**–**1.25)	0.99 (0.88–1.12)
No substance abuse	1,705 (20.79)	1	1	1	1
Behavioral and/or emotional disorders (ICD-10: F91–F98)	55 (24.55)	1.16 (0.89**–**1.52)	1.24 (0.94**–**1.62)	1.15 (0.88**–**1.50)	1.00 (0.76–1.31)
No behavioral nor emotional disorders	2,020 (21.28)	1	1	1	1
Schizophrenia/psychoses (ICD-10: F20–F29)	59 (45.74)	**2.60 (2.01–3.37)**	**2.57 (1.98–3.33)**	**2.35 (1.81–3.05)**	**2.16 (1.66–2.80)**
No schizophrenia/psychoses	2,056 (21.29)	1	1	1	1
Mental retardation^c^ (ICD-10: F70–F83, F85-F89)	19 (32.20)	**1.72 (1.10–2.70)**	**1.64 (1.05–2.58)**	**1.85 (1.18–2.91)**	**1.65 (1.05–2.61)**
No mental retardation	2,016 (21.02)	1	1	1	1
Other mental disorders (ICD-10: Other F)	314 (31.06)	**1.65 (1.47–1.86)**	**1.65 (1.46–1.86)**	**1.51 (1.34–1.71)**	**1.32 (1.16–1.50)**
No other mental disorders	1,761 (20.23)	1	1	1	1
Musculoskeletal disorders (ICD-10: M00–M99)	108 (22.04)	1.05 (0.87**–**1.28)	1.09 (0.90**–**1.33)	0.95 (0.78**–**1.16)	0.95 (0.78–1.15)
No musculoskeletal disorders	1,967 (21.32)	1	1	1	1
Other somatic disorders (ICD-10: all remaining codes)	1,250 (20.24)	**1.18 (1.08–1.28)**	1.06 (0.96**–**1.15)	1.02 (0.94**–**1.12)	0.99 (0.90–1.08)
No other somatic disorders	1,119 (20.48)	1	1	1	1

a Adjusted for sex, age, and educational level by matching, as well as for family composition, type of living area and region of birth.

b As Model 1 and additionally adjusted for baseline unemployment and baseline sickness absence.

c Mutually adjusted for all the other variables.

d <10 years is equivalent to elementary school; 10 to 12 years is equivalent to upper and secondary school; and >12 years is equivalent to college/university level.

e Big cities included: Stockholm, Göteborg and Malmö; medium-sized cities: cities with more than 90,000 inhabitants within 30 km distance from the center of the city; otherwise small cities/villages.

Estimates in bold indicate significant statistical significance (p < 0.05).

People who did not have any previous income from work had a more than 2.5-fold risk of receiving disability pension (HR = 2.64, 95% CI 2.35–2.98), compared with those with some income from work before diagnosis, in the fully adjusted model (Table 2). Compared with people with no unemployment during baseline, those who were unemployed for 0 to 6 months at baseline had a slightly lower risk of disability pension (HR = 0.84, 95% CI 0.76–0.93). A 2.5-fold risk of disability pension was seen in people who had >90 days of sickness absence 1 year before CED (HR = 2.48, 95% CI 2.17–2.83), compared with those without any registered sickness absence.

Having schizophrenia/psychoses (HR = 2.16, 95%CI 1.66–2.80), autism-spectrum disorders (HR = 1.87, 95%CI 1.42–2.46), anxiety and/or stress-related disorders (HR = 1.34, 95%CI 1.22–1.49), and depression and/or bipolar disorders (HR = 1.22, 95%CI 1.09–1.36), were associated with significantly increased risk of disability pension (Table 2).

Similar tendencies for the associations between most of the covariates and disability pension were presented in both genders (Table 3). Men diagnosed with ADHD and who were married/cohabiting with children in the household, had a significantly decreased risk of disability pension (HR = 0.61, 95%CI 0.43–0.86), compared with those who were single and without children. The corresponding risk was similar among women of different family compositions. Although having no income from work at baseline increased the risk of disability pension in both men and women, the risk estimates were, however, slightly higher for men (HR = 3.01, 95%CI 2.53–3.59) compared with women (HR = 2.34, 95%CI 1.99–2.75). The same tendency was also seen for having sickness absence at baseline.

**Table 3.** Hazard Ratios (HRs) with 95% Confidence Interval (CI) for Disability Pension in Association with Sociodemographic, Work-Related Factors and Comorbid Diagnoses among Male and Female Patients with Diagnosed ADHD, Respectively.

	Male (*n* = 5,552)			Female (*n* = 4,166)		
	Number of DP (% of all people in the corresponding stratum)	Crude model	Adjusted model^a^	Number of DP (% of all people in the corresponding stratum)	Crude model	Adjusted model^a^
	*n* (%)	HR (95% CI)	HR (95% CI)	*n* (%)	HR (95% CI)	
Sociodemographic factors						
Age at diagnosis						
19–24 years (young)	726 (21.83)	**1.63 (1.43–1.86)**	**1.60 (1.39–1.84)**	763 (28.13)	**1.56 (1.36–1.79)**	**1.47 (1.26–1.72)**
25–29 years (old)	311 (13.96)	1	1	275 (18.91)	1	1
Educational level (years)^c^						
Low (<10)	634 (22.51)	**2.22 (1.61–3.07)**	**1.97 (1.42–2.74)**	570 (30.83)	**2.70 (2.08–3.50)**	**2.04 (1.55–2.67)**
Medium (10–12)	364 (15.28)	**1.43 (1.03–1.99)**	**1.45 (1.04–2.03)**	405 (22.14)	**1.82 (1.39–2.37)**	**1.53 (1.16–2.01)**
High (>12)	39 (11.02)	1	1	63 (12.91)	1	1
Region of birth						
Sweden	947 (18.68)	1	1	965 (24.88)	1	1
Nordic countries	8 (15.38)	N/A^b^	N/A	6 (28.57)	N/A	N/A
EU27	15 (22.39)	1.23 (0.74, 2.05)	1.26 (0.76**–**2.11)	5 (15.15)	N/A	N/A
Rest of world	67 (18.41)	1.00 (0.78, 1.28)	0.95 (0.74**–**1.22)	62 (26.61)	1.09 (0.84**–**1.41)	1.19 (0.91**–**1.54)
Family composition						
Married/cohabiting without children	9 (21.95)	N/A	N/A	22 (29.33)	1.23 (0.81**–**1.88)	1.26 (0.82**–**1.93)
Married/cohabiting with children	33 (9.24)	**0.44 (0.31–0.63)**	**0.61 (0.43–0.86)**	79 (20.41)	**0.80 (0.61–0.97)**	**0.81 (0.64–1.03)**
Single without children	991 (19.34)	1	1	810 (25.35)	1	1
Single with children	4 (12.90)	N/A	N/A	127 (24.95)	0.98 (0.81**–**1.18)	1.00 (0.82**–**1.22)
Type of living area^d^						
Big cities	308 (17.00)	1	1	329 (21.69)	1	1
Medium-sized cities	400 (19.67)	**1.16 (1.00–1.35)**	1.06 (0.92**–**1.24)	379 (24.72)	**1.16 (1.00–1.35)**	1.01 (0.87**–**1.18)
Small towns	329 (19.28)	1.14 (0.98**–**1.33)	1.00 (0.85**–**1.17)	330 (29.57)	**1.43 (1.23–1.67)**	**1.20 (1.02–1.40)**
Work-related factors						
Income from work						
Yes	176 (9.06)	1	1	219 (14.48)	1	1
No	861 (23.85)	**2.92 (2.48–3.43)**	**3.01 (2.53–3.59)**	819 (30.86)	**2.39 (2.06–2.78)**	**2.34 (1.99–2.75)**
Unemployment at baseline						
0 days	492 (18.14)	1	1	564 (23.38)	1	1
1–180 days (short)	366 (17.76)	0.97 (0.85**–**1.11)	**0.81 (0.70–0.92)**	315 (24.29)	1.04 (0.90**–**1.19)	0.88 (0.76**–**1.01)
>180 days (long)	179 (22.98)	**1.30 (1.10–1.55)**	0.92 (0.77**–**1.10)	159 (34.79)	**1.60 (1.34–1.91)**	1.14 (0.95**–**1.36)
Sickness absence at baseline						
0 days	854 (17.97)	1	1	800 (23.61)	1	1
1–90 days (short)	37 (10.66)	**0.57 (0.41–0.79)**	**0.96 (0.68–1.34)**	60 (19.17)	0.79 (0.60**–**1.02)	1.22 (0.93**–**1.61)
>90 days (long)	146 (32.30)	**1.99 (1.67–2.37)**	**2.72 (2.24–3.31)**	178 (38.36)	**1.81 (1.53–2.12)**	**2.23 (1.86–2.68)**
Comorbid diagnoses						
Depression and/or bipolar disorders (ICD-10^b^ F30-F34)	213 (27.06)	**1.65 (1.42–1.92)**	**1.37 (1.17–1.62)**	264 (29.30)	**1.30 (1.13–1.49)**	1.12 (0.96–1.30)
No depression nor bipolar disorders	824 (17.29)	1	1	774 (23.71)	1	1
Anxiety and/or stress-related disorders (ICD-10: F40-F48)	276 (26.74)	**1.70 (1.48–1.95)**	**1.45 (1.25–1.68)**	346 (30.84)	**1.44 (1.27–1.64)**	**1.26 (1.10–1.44)**
No anxiety nor stress-related disorders	761 (16.84)	1	1	692 (22.73)	1	1
Autism-spectrum disorders (ICD-10: F84)	37 (39.36)	**2.54 (1.83–3.53)**	**2.33 (1.66–3.27)**	17 (34.00)	1.50 (0.93–2.43)	1.37 (0.84–2.22)
No autism-spectrum disorders	1,000 (18.32)	1	1	1,021 (24.81)	1	1
Substance abuse (ICD-10: F10-F19)	225 (22.26)	1.30 (1.13**–**1.51)	1.04 (0.89**–**1.22)	145 (28.60)	**1.23 (1.04–1.47)**	0.92 (0.77**–**1.11)
No substance abuse	812 (17.88)	1	1	893 (24.41)	1	1
Behavioral and/or emotional disorders (ICD-10: F91-F98)	31 (21.83)	1.20 (0.84**–**1.71)	0.97 (0.67**–**1.41)	24 (29.27)	1.18 (0.78**–**1.76)	1.00 (0.66**–**1.50)
No behavioral nor emotional disorders	1,006 (18.60)	1	1	1,014 (24.83)	1	1
Schizophrenia/psychoses (ICD-10: F20-F29)	38 (43.68)	**2.82 (2.04–3.90)**	**2.40 (1.73–3.33)**	21 (50.00)	**2.52 (1.64–3.88)**	**1.84 (1.18–2.87)**
No schizophrenia/psychoses	999 (18.28)	1	1	1,017 (24.66)	1	1
Mental retardation (ICD-10: F70-F83, F85-F89)	12 (33.33)	**2.03 (1.15–3.58)**	**1.93 (1.07–3.47)**	7 (30.43)	N/A	N/A
No mental retardation	1,025 (18.58)	1	1	1,031 (24.89)	1	1
Other mental disorders (ICD-10: Other F)	105 (25.80)	**1.50 (1.23–1.84)**	1.20 (0.97**–**1.48)	209 (34.60)	**1.62 (1.39–1.88)**	**1.44 (1.22–1.69)**
No other mental disorders	932 (18.11)	1	1	829 (23.27)	1	1
Musculoskeletal disorders (ICD-10: M00-M99)	44 (15.71)	0.83 (0.62**–**1.12)	0.75 (0.55**–**1.01)	64 (30.48)	**1.30 (1.01–1.67)**	1.15 (0.88**–**1.49)
No musculoskeletal disorders	993 (18.84)	1	1	974 (24.62)	1	1
Other somatic disorders (ICD-10: all remaining codes^d^)	398 (18.87)	1.03 (0.91**–**1.16)	0.95 (0.84**–**1.08)	558 (26.03)	1.12 (0.99**–**1.26)	1.01 (0.90**–**1.15)
No other somatic disorders	639 (18.56)	1	1	480 (23.74)	1	1

a Mutually adjusted for all the other variables.

b When the number of people who had the outcome (disability pension) in the specific stratum was lower than 10, the results were shown as not applicable (N/A).

c <10 years is equivalent to elementary school; 10 to 12 years is equivalent to upper and secondary school; and >12 years is equivalent to college/university level.

d Big cities included: Stockholm, Göteborg and Malmö; medium-sized cities: cities with more than 90,000 inhabitants within 30 km distance from the center of the city; otherwise small cities/villages.

Estimates in bold indicate significant statistical significance (p < 0.05).

Men with depression and/or bipolar disorders at baseline had a nearly 40% increased risk of disability pension compared with those without these disorders, whereas the risks were similar between women with and without such diagnoses (Table 3). Having autism-spectrum disorders at baseline also doubled men’s risk of receiving disability pension (HR = 2.33, 95%CI 1.66–3.27), however, the increased risk was not observed in women. An elevated risk of disability pension associated with other mental disorders was found in women (HR = 1.44, 95%CI 1.22–1.69), but not in men. The risk of disability pension was more evident among men with comorbid schizophrenia/psychoses (HR = 2.40, 1.73–3.33) than among women (HR = 1.84, 95%CI 1.18–2.87).

## Discussion

In this longitudinal population-based prospective cohort study on 9,718 young adults diagnosed with ADHD, 21% of them received disability pension during the 5 years’ follow-up. An increased risk of future disability pension was associated with female sex, younger age at diagnosis, low educational level, without income from work at baseline, with long period of sickness absence at baseline, and comorbid mental disorders (schizophrenia/psychoses, autism-spectrum disorders, anxiety, and/or stress-related disorders, and depression, and/or bipolar disorders).

Both the incidence and prevalence of ADHD has increased in young women in Sweden, even though the majority of the young adults with ADHD diagnosis consists of men (Giacobini et al., 2018; Polyzoi et al., 2018). Currently, we noted a 23% increased risk of having disability pension in young women diagnosed with ADHD, in comparison with men diagnosed with ADHD. In general, women are more likely to be granted disability pension than men (Haukenes et al., 2012; Karlsson et al., 2006), which also was consistent with our results. Specifically, our finding was in line with two other studies on adults diagnosed with ADHD: a Swedish study reported a 30% increased risk of sickness absence and disability pension during a 3-year follow-up in women with ADHD diagnosis compared with men (Virtanen et al., 2020), while a Norwegian study found a doubled risk of work disability among women with ADHD diagnosis in comparison with men, during an 1-year observation (Fredriksen et al., 2014). Additionally, in terms of presented ADHD symptoms, women tend to internalize problems and have more intellectual impairments than men (Gershon & Gershon, 2002), which may also pose a challenge for these women to remain in the workforce. Conclusively, special attention to the long-term well-being and work outcomes of these women is called for.

Interestingly, we noted an elevated risk of having disability pension in patients diagnosed at younger age (19–24 years) compared with those diagnosed at older age (25–29 years), which was in contrast with a previous study (Virtanen et al., 2020). One of the plausible explanations may be that individuals diagnosed at a younger age may have more troubles caused by ADHD symptoms. These symptoms such as hyperactivity, inattention and impulsivity may hamper their life, particularly regarding education. Due to potential disadvantages in educational attainment, these individuals may later be more likely to be excluded from the labor market. Most of the research has so far only investigated individuals diagnosed with ADHD before adulthood (Virtanen et al., 2020), which has led us to speculate a different long-term work impact of the disease in young adults with the same diagnosis. Future studies are warranted for a deeper understanding of these aspects.

In our study, work-related factors, such as having no income from work and having sickness absence over 90 days during the year before CED, were associated with an increase in the risk of future disability pension. One possible explanation is that individuals with long-term sickness absence tended to be in the process for disability pension (Karlsson et al., 2008) and therefore were more likely to be have disability pension later. Even though previous studies have reported higher occurrence of work disability and workplace difficulties among patients with an ADHD diagnosis (de Graaf et al., 2008; Halmoy et al., 2009; Küpper et al., 2012; Virtanen et al., 2020), none has investigated the association between work-related factors at baseline and longitudinal work outcomes in these individuals. Stratifying by sex, we further confirmed a similar tendency between a lower labor market involvement and a higher risk of disability pension, in both men and women. However, the association seemed to be slightly more considerable in men than women. Whether or not it reflected a true gender impact, more studies are needed in the future.

Comorbid mental disorders are highly related to ADHD, in terms of etiology (Gnanavel et al., 2019; Katzman et al., 2017). The complicated nature of ADHD and its relationship with other mental disorders highlight the importance of elucidating the associations between ADHD, comorbid mental disorders and work disability among adult patients. Currently, we noted that some comorbid disorders, were highly associated with the risk of granted disability pension. Similar findings have been presented by an earlier Swedish study, stating that patients with an ADHD diagnosis with comorbid mental disorders had a nearly 60% risk increase of having sickness absence and disability pension days than those without these comorbidities during a 3-year follow-up (Virtanen et al., 2020). Although the previous study did not distinguish the subtypes of comorbid mental disorders and adjusted for only the presence of them as a dichotomous variable, we were able to further differentiate the various impact of specific diagnoses in the study.

To illustrate the subtypes of comorbid mental disorders is critical in designing targeted intervention programs to prevent potential work disability related to specific subtypes. In general, ADHD has been shown to have a negative impact on one’s attention and cognitive ability (Giacobini et al., 2018), while it also interacts with patients’ comorbid mental disorders and other characteristics. Depression and anxiety, as types of common mental disorders, have been associated with an increased risk of work disability in adult ADHD patients (Fredriksen et al., 2014; Helgesson et al., 2018). Similarly, among individuals diagnosed with ADHD, we found a 20% higher risk of having disability pension in those with comorbidities of depression and bipolar disorders, and a 35% higher risk in patients with anxiety and stress-related disorders. These findings call for awareness of comorbid mental disorders in the course of work ability rehabilitation among young adults with ADHD diagnosis.

In the sex-stratified analyses, we found higher risk estimates of disability pension among men with depression and bipolar disorders than among women with such comorbidities. Men and women seem to perceive depression differently (Möller-Leimkühler, 2002), which may likely lead to different work disability outcomes. A Brazilian study based on an occupational setting showed that men workers with depression tended to have more cognitive dysfunctions than women, while men also took lengthier period of time off work due to depression (Wang & Gorenstein, 2015). Nevertheless, knowledge in gender differences in patients with ADHD with depression and bipolar as well as comorbid diagnoses is lacking. The potential elevated risk of work disability in men diagnosed with ADHD and other comorbid mental disorders reported by our study should therefore be further elucidated.

Of all the studied comorbid mental disorders, schizophrenia/psychoses rendered the highest risk associated with receiving future disability pension in patients with ADHD diagnosis—about a 2-fold risk compared with those patients with ADHD diagnosis without such disorders. Additionally, the increased risk was observed in both sexes. Schizophrenia and psychoses as severe mental disorders have been associated with high rates of both unemployment and work disability (Holm et al., 2021; Marwaha et al., 2007; Salkever et al., 2007). Targeted vocational interventions for them have been found effective in achieving higher work involvement (Falkum et al., 2017; Tsang et al., 2010). Therefore, it is important that young individuals with ADHD diagnosis and schizophrenia/psychoses as comorbid diagnoses are given extra attention regarding future work outcomes.

### Strengths and Limitations

One of the most prominent strengths of our study is the use of multiple longitudinal population-based Swedish registers. These registers contain high-quality data covering information on sociodemographic, work- and health-related variables for the study population, with long follow-up possibilities. All the data used were administrative, instead of self-reported information that is often hampered by misclassification. The use of these data further ensured the quality of the information acquired. Besides, our study took the advantage of complete registration of the relevant information in the Swedish labor market which has high work participation (Gottfries, 2018).

Several limitations were also noted in the study, which warrant consideration. The diagnosis of incident ADHD was only based on the specialized healthcare visits that derived from the National Patient Register, without data from the primary healthcare. However, according to the recommendation of ADHD clinical care guideline (Wolraich et al., 2019), the diagnosis of ADHD should be ascertained at specialized health care. Thus, the use of only specialized health care to capture ADHD diagnosis may not have affected the study results much. Further, we could not measure age at onset due to the limitation of data sources, that is, we did not have access to complete data retrospectively. Secondly, the first 14 days of sickness absence were paid by the employers and were not included in the data. However, given the study outcome, that is, as disability pension was independent of the first 14 days of sickness absence, the lack of this information would not have affected our findings. Thirdly, we did not have access to data on the severity of ADHD. Future studies with such clinical information on the disease are warranted.

## Conclusion

Special attention should be given to early vocational rehabilitation of patients diagnosed with ADHD as young adults, in order to prevent future long-term marginalization from the labor market. Risk factors such as previous low involvement in the labor market, common comorbid mental disorders such as depression, bipolar disorders, anxiety, and stress-related disorders need to be monitored for young adults with ADHD diagnosis.

## References

[bibr1-10870547211025605] Brooke H. L. Talback M. Hornblad J. Johansson L. A. Ludvigsson J. F. Druid H. Feychting M. Ljung R. (2017). The Swedish cause of death register. European Journal of Epidemiology, 32(9), 765–773. 28983736 10.1007/s10654-017-0316-1 PMC5662659

[bibr2-10870547211025605] Chung W. Jiang S. F. Paksarian D. Nikolaidis A. Castellanos F. X. Merikangas K. R. Milham M. P. (2019). Trends in the prevalence and incidence of attention-deficit/hyperactivity disorder among adults and children of different racial and ethnic groups. JAMA Network Open, 2(11), e1914344. 31675080 10.1001/jamanetworkopen.2019.14344 PMC6826640

[bibr3-10870547211025605] Coghill D. R. Banaschewski T. Soutullo C. Cottingham M. G. Zuddas A. (2017). Systematic review of quality of life and functional outcomes in randomized placebo-controlled studies of medications for attention-deficit/hyperactivity disorder. European Child & Adolescent Psychiatry, 26(11), 1283–1307. 28429134 10.1007/s00787-017-0986-y PMC5656703

[bibr4-10870547211025605] Cortese S. Coghill D. (2018). Twenty years of research on attention-deficit/hyperactivity disorder (ADHD): Looking back, looking forward. Evidence-Based Mental Health, 21(4), 173–176. 30301823 10.1136/ebmental-2018-300050 PMC10270437

[bibr5-10870547211025605] de Graaf R. Kessler R. C. Fayyad J. ten Have M. Alonso J. Angermeyer M. Borges G. Demyttenaere K. Gasquet I. de Girolamo G. Haro J. M. Jin R. Karam E. G. Ormel J. Posada-Villa J . (2008). The prevalence and effects of adult attention-deficit/hyperactivity disorder (ADHD) on the performance of workers: Results from the WHO World Mental Health Survey Initiative. Occupational and Environmental Medicine, 65(12), 835–842. 18505771 10.1136/oem.2007.038448 PMC2665789

[bibr6-10870547211025605] Falkum E. Klungsøyr O. Lystad J. U. Bull H. C. Evensen S. Martinsen E. W. Friis S. Ueland T. (2017). Vocational rehabilitation for adults with psychotic disorders in a Scandinavian welfare society. BMC Psychiatry, 17(1), 24. 28095813 10.1186/s12888-016-1183-0 PMC5240414

[bibr7-10870547211025605] Fayyad J. Sampson N. A. Hwang I. Adamowski T. Aguilar-Gaxiola S. Al-Hamzawi A. Andrade L. H. S. G. Borges G. de Girolamo G. Florescu S. Gureje O. Haro J. M. Hu C. Karam E. G. Lee S. Navarro-Mateu F. O’Neill S. Pennell B.-E. Piazza M. . . . Kessler R. C. (2017). The descriptive epidemiology of DSM-IV Adult ADHD in the World Health Organization World Mental Health Surveys. ADHD Attention Deficit and Hyperactivity Disorders, 9(1), 47–65. 27866355 10.1007/s12402-016-0208-3 PMC5325787

[bibr8-10870547211025605] Fredriksen M. Dahl A. A. Martinsen E. W. Klungsoyr O. Faraone S. V. Peleikis D. E. (2014). Childhood and persistent ADHD symptoms associated with educational failure and long-term occupational disability in adult ADHD. ADHD Attention Deficit and Hyperactivity Disorders, 6(2), 87–99. 24497125 10.1007/s12402-014-0126-1 PMC4033786

[bibr9-10870547211025605] Gershon J. Gershon J. (2002). A meta-analytic review of gender differences in ADHD. Journal of Attention Disorders, 5(3), 143–154. 11911007 10.1177/108705470200500302

[bibr10-10870547211025605] Giacobini M. Medin E. Ahnemark E. Russo L. J. Carlqvist P. (2018). Prevalence, patient characteristics, and pharmacological treatment of children, adolescents, and adults diagnosed with ADHD in Sweden. Journal of Attention Disorders, 22(1), 3–13. 25376193 10.1177/1087054714554617

[bibr11-10870547211025605] Gnanavel S. Sharma P. Kaushal P. Hussain S. (2019). Attention deficit hyperactivity disorder and comorbidity: A review of literature. World Journal of Clinical Cases, 7(17), 2420–2426. 31559278 10.12998/wjcc.v7.i17.2420 PMC6745333

[bibr12-10870547211025605] Gottfries N. (2018). The labor market in Sweden since the 1990s. IZA World of Labor, 2018: 411.

[bibr13-10870547211025605] Halmoy A. Fasmer O. B. Gillberg C. Haavik J. (2009). Occupational outcome in adult ADHD: Impact of symptom profile, comorbid psychiatric problems, and treatment: A cross-sectional study of 414 clinically diagnosed adult ADHD patients. Journal of Attention Disorders, 13(2), 175–187. 19372500 10.1177/1087054708329777

[bibr14-10870547211025605] Haukenes I. Gjesdal S. Rortveit G. Riise T. Maeland J. G. (2012). Women’s higher likelihood of disability pension: the role of health, family and work. A 5-7 years follow-up of the Hordaland Health Study. BMC Public Health, 12, 720. 22943493 10.1186/1471-2458-12-720 PMC3508825

[bibr15-10870547211025605] Helgesson M. Tinghog P. Niederkrotenthaler T. Saboonchi F. Mittendorfer-Rutz E. (2017). Labour-market marginalisation after mental disorders among young natives and immigrants living in Sweden. BMC Public Health, 17(1), 593. 28645250 10.1186/s12889-017-4504-4 PMC5481931

[bibr16-10870547211025605] Helgesson M. Tinghog P. Wang M. Rahman S. Saboonchi F. Mittendorfer-Rutz E. (2018). Trajectories of work disability and unemployment among young adults with common mental disorders. BMC Public Health, 18(1), 1228. 30400785 10.1186/s12889-018-6141-y PMC6219052

[bibr17-10870547211025605] Holm M. Taipale H. Tanskanen A. Tiihonen J. Mitterdorfer-Rutz E. (2021). Employment among people with schizophrenia or bipolar disorder: A population-based study using nationwide registers. Acta Psychiatrica Scandinavica, 143(1), 61–71. 33155273 10.1111/acps.13254 PMC7839734

[bibr18-10870547211025605] Karlsson N. E. Carstensen J. M. Gjesdal S. Alexanderson K. A. (2008). Risk factors for disability pension in a population-based cohort of men and women on long-term sick leave in Sweden. European Journal of Public Health, 18(3), 224–231. 18245150 10.1093/eurpub/ckm128

[bibr19-10870547211025605] Karlsson N. Borg K. Carstensen J. Hensing G. Alexanderson K. (2006). Risk of disability pension in relation to gender and age in a Swedish county; a 12-year population based, prospective cohort study. Work, 27(2), 173–179. 16971764

[bibr20-10870547211025605] Katzman M. A. Bilkey T. S. Chokka P. R. Fallu A. Klassen L. J. (2017). Adult ADHD and comorbid disorders: Clinical implications of a dimensional approach. BMC Psychiatry, 17(1), 302. 28830387 10.1186/s12888-017-1463-3 PMC5567978

[bibr21-10870547211025605] Küpper T. Haavik J. Drexler H. Ramos-Quiroga J. A. Wermelskirchen D. Prutz C. Schauble B. (2012). The negative impact of attention-deficit/hyperactivity disorder on occupational health in adults and adolescents. International Archives of Occupational and Environmental Health, 85(8), 837–847. 22752312 10.1007/s00420-012-0794-0

[bibr22-10870547211025605] Laaksonen M. Mastekaasa A. Martikainen P. Rahkonen O. Piha K. Lahelma E. (2010). Gender differences in sickness absence–the contribution of occupation and workplace. Scandinavian Journal of Work, Environment & Health, 36(5), 394–403. 10.5271/sjweh.2909 20213051

[bibr23-10870547211025605] Ludvigsson J. F. Andersson E. Ekbom A. Feychting M. Kim J. L. Reuterwall C. Heurgren M. Olausson P. O. (2011). External review and validation of the Swedish national inpatient register. BMC Public Health, 11, 450. 21658213 10.1186/1471-2458-11-450 PMC3142234

[bibr24-10870547211025605] Ludvigsson J. F. Svedberg P. Olen O. Bruze G. Neovius M. (2019). The longitudinal integrated database for health insurance and labour market studies (LISA) and its use in medical research. European Journal of Epidemiology, 34(4), 423–437. 30929112 10.1007/s10654-019-00511-8 PMC6451717

[bibr25-10870547211025605] Ludvigsson J. Otterblad-Olausson P. Pettersson B. Ekbom A. (2009). The Swedish personal identity number: Possibilities and pitfalls in healthcare and medical research. European Journal of Epidemiology, 24(11), 659–667. 19504049 10.1007/s10654-009-9350-y PMC2773709

[bibr26-10870547211025605] Marwaha S. Johnson S. Bebbington P. Stafford M. Angermeyer M. C. Brugha T. Azorin J. M. Kilian R. Hansen K. Toumi M. (2007). Rates and correlates of employment in people with schizophrenia in the UK, France and Germany. British Journal of Psychiatry, 191, 30–37. 10.1192/bjp.bp.105.020982 17602122

[bibr27-10870547211025605] Möller-Leimkühler A. M. (2002). Barriers to help-seeking by men: A review of sociocultural and clinical literature with particular reference to depression. Journal of Affective Disorders, 71(1–3), 1–9. 12167495 10.1016/s0165-0327(01)00379-2

[bibr28-10870547211025605] Österlund N. (2011). MiDAS - sjukpenning och rehabiliteringspenning (MiDAS - sickness benefit and rehabilitation allowance). Swedish Social Insurance Agency.

[bibr29-10870547211025605] Polyzoi M. Ahnemark E. Medin E. Ginsberg Y. (2018). Estimated prevalence and incidence of diagnosed ADHD and health care utilization in adults in Sweden - a longitudinal population-based register study. Neuropsychiatric Disease and Treatment, 14, 1149–1161. 29765219 10.2147/NDT.S155838 PMC5944447

[bibr30-10870547211025605] Rydell M. Lundström S. Gillberg C. Lichtenstein P. Larsson H. (2018). Has the attention deficit hyperactivity disorder phenotype become more common in children between 2004 and 2014? Trends over 10 years from a Swedish general population sample. Journal of Child Psychology and Psychiatry, 59(8), 863–871. 29484650 10.1111/jcpp.12882

[bibr31-10870547211025605] Salkever D. S. Karakus M. C. Slade E. P. Harding C. M. Hough R. L. Rosenheck R. A. Swartz M. S. Barrio C. Yamada A. M. (2007). Measures and predictors of community-based employment and earnings of persons with schizophrenia in a multisite study. Psychiatric Services, 58(3), 315–324. 17325103 10.1176/ps.2007.58.3.315

[bibr32-10870547211025605] Sobanski E. (2006). Psychiatric comorbidity in adults with attention-deficit/hyperactivity disorder (ADHD). European Archives of Psychiatry and Clinical Neuroscience, 256(Suppl. 1), i26–i31. 10.1007/s00406-006-1004-4 16977548

[bibr33-10870547211025605] Swedish Social Insurance Agency. (2012). Tio år med aktivitetsersättning- en studie av situationen för unga med aktivitetsersättning på grund av nedsatt arbetsförmåga (Ten years with activity compensation - a study on the situation among young adults with activity compensation due to work disability) [In Swedish].

[bibr34-10870547211025605] Thapar A. Cooper M. (2016). Attention deficit hyperactivity disorder. The Lancet, 387(10024), 1240–1250. 10.1016/S0140-6736(15)00238-X 26386541

[bibr35-10870547211025605] Thomas R. Sanders S. Doust J. Beller E. Glasziou P. (2015). Prevalence of attention-deficit/hyperactivity disorder: A systematic review and meta-analysis. Pediatrics, 135(4), e994–e1001. 25733754 10.1542/peds.2014-3482

[bibr36-10870547211025605] Tsang H. W. Leung A. Y. Chung R. C. Bell M. Cheung W. M. (2010). Review on vocational predictors: A systematic review of predictors of vocational outcomes among individuals with schizophrenia: An update since 1998. The Australian and New Zealand Journal of Psychiatry, 44(6), 495–504. 20482409 10.3109/00048671003785716

[bibr37-10870547211025605] Virtanen M. Lallukka T. Kivimaki M. Alexanderson K. Ervasti J. Mittendorfer-Rutz E. (2020). Neurodevelopmental disorders among young adults and the risk of sickness absence and disability pension: A nationwide register linkage study. Scandinavian Journal of Work, Environment & Health, 46(4), 410–416. 10.5271/sjweh.3888 PMC8506319 32076730

[bibr38-10870547211025605] Wang Y. P. Gorenstein C. (2015). Gender differences and disabilities of perceived depression in the workplace. Journal of Affective Disorders, 176, 48–55. 25699670 10.1016/j.jad.2015.01.058

[bibr39-10870547211025605] Wettermark B. Hammar N. Fored C. M. Leimanis A. Olausson P. O. Bergman U. Persson I. Sundström A. Westerholm B. Rosén M. (2007). The new Swedish Prescribed Drug Register–opportunities for pharmacoepidemiological research and experience from the first six months. Pharmacoepidemiology and Drug Safety, 16(7), 726–735. 16897791 10.1002/pds.1294

[bibr40-10870547211025605] Wolraich M. L. Hagan J. F. Jr. Allan C. Chan E. Davison D. Earls M. Evans S. W. Flinn S. K. Froehlich T. Frost J. Holbrook J. R. (2019). Clinical practice guideline for the diagnosis, evaluation, and treatment of attention-deficit/hyperactivity disorder in children and adolescents. Pediatrics, 144(4). https://pediatrics.aappublications.org/content/144/4/e20192528 10.1542/peds.2019-2528 PMC7067282 31570648

